# An Essay on the Fevers Which Prevailed in Natchez in 1837, ’8 and ’9

**Published:** 1840-06

**Authors:** Samuel Hogg


### WESTERN JOURNAL

OF

### MEDICINE AND SURGERY.

JUNE, 1840.

### Art. I.—*An account of the Epidemic Fevers of Natchez, Mississippi, in the years* 1837–'8–'9. By Samuel Hogg, M. D.

Not only the medical profession, but the public in general, have a claim on the physicians of Natchez for at least a passing notice of the malignant epidemics which have scourged this devoted city during the years 1837–'8–'9.

No one of the faculty having undertaken the task, I deem it my duty, however feeble my efforts may prove, to give to the public such information on this interesting subject as my limited means will enable me, hoping that my brethren will take the remarks for what they may be worth and will withhold their criticism.

In advance of the observations proposed to be made on the two epidemics, it may be proper to give a topographical description of the city of Natchez, the character of the seasons, and a general view of the diseases immediately preceding and subsequent to them, together with a general account of their treatment.

Many of the facts upon which these observations are founded have come within the knowledge of the writer, the others have been communicated by medical men of the first respectability and may be relied upon.

Natchez is situated on a commanding bluff, which forms the east bank of the Mississippi river, in latitude thirty-one degrees thirty-four minutes, and longitude ninety-one degrees twenty-four minutes, west from Greenwich, and fourteen degrees twenty-three seconds west of Washington city. It is laid off in regular squares, intersected by streets of moderate width and drained by sewers. The site of the city was originally undulating, with a delightful landscape extending back south and east—the country around for many miles is much injured by large gullies or bayous—the city is almost surrounded by them.

In 1834 the citizens commenced extensive graduations, removing the hills and filling the bayous, of which there were many—great improvements in building also commenced, which not only brought in much lumber, but made almost all the surrounding country mortar-beds or brick-yards. The population increased rapidly, principally composed of the laboring classes, stout, athletic and unacclimated, and the number of inhabitants in 1837 exceeded six thousand.

What is known as "under the hill at Natchez" extends from the termination of the bluff to the margin of the river, a distance of from one to two hundred yards in width, and from a half to three-fourths of a mile in length up the river—its fixed population is between five and six hundred.

On the west side of the river the country is low and level, intersected by bayous, lake Concordia and other smaller lakes, which seem to have been at some time the bed of the river. There are now many large cotton plantations on that side in a high state of cultivation, and the blacks are generally healthy and seem contented. The city is supplied with good provisions, excellent cistern water, and ice in the summer. The citizens generally were temperate, orderly and industrious in their different occupations during those years.

The weather in 1836 was not in the extreme, either in temperature or in rain, the highest rise of the thermometer was 92° for a few days only at 3 o'clock, P. M., in July and August. The lowest was 24° for two or three days in November and December—there was no snow during the year.

There were 113 days on which it rained and 63 days on which there was lightning and thunder; much rain fell previously to the month of August, and the Spring months were disagreeable.

From the following meteorological register of the years 1837–'8–'9 I am indebted to our distinguished fellow-citizen and accurate observer, Dr. Henry Tooley, and deem it sufficiently important to give it in full:

### METEOROLOGICAL REGISTER KEPT AT NATCHEZ, 1837.

| 1837. | *Thermometer.* 6 A. M. | 12 M. | 6 P. M. | *Barometer.* | | | cl.days | cl'dy. | rain | thaw | fog. | snow. |
|---|---|---|---|---|---|---|---|---|---|---|---|---|
| January, | 42,5 | 48 | 51,8 | 29,76 | 29,78 | 29,72 | 11 | 7 | 9 | 1 | 2 | 2 |
| February, | 43 | 53 | 57 | 29,72 | 29,65 | 29,69 | 8 | 8 | 10 | 2 | 2 | 0 |
| March, | 51,8 | 61 | 64 | 29,70 | 29,66 | 29,69 | 7 | 7 | 12 | 0 | 5 | 0 |
| April, | 57 | 65 | 71 | 29,72,8 | 29,71 | 29,67 | 11 | 14 | 5 | 3 | 0 | 0 |
| May, | 65 | 75 | 79 | 29,72,2 | 29,71,3 | 29,65 | 23 | 4 | 4 | 5 | 0 | 0 |
| June, | 74,2 | 82,1 | 86 | 29,66,9 | 29,71,2 | 29,66 | 5 | 18 | 7 | 0 | 0 | 0 |
| July, | 76,5 | 84,3 | 88,3 | 29,74,7 | 29,77,1 | 29,74,1 | 0 | 21 | 10 | 11 | 0 | 0 |
| August, | 75,1 | 84,9 | 87,1 | 29,74,1 | 29,75,8 | 29,73,2 | 0 | 25 | 6 | 1 | 0 | 0 |
| September | 71,1 | 73,5 | 87,6 | 29,77,3 | 29,78,1 | 29,74 | 4 | 16 | 10 | 3 | 0 | 0 |
| October, | 61,8 | 68,7 | 72,2 | 29,86,4 | 29,91,1 | 29,87,6 | 8 | 18 | 5 | 2 | 0 | 0 |
| November | 53,2 | 64,1 | 72,2 | 29,81,2 | 29,84,1 | 29,81 | 11 | 13 | 6 | 0 | 0 | 0 |
| December | 46,6 | 55,6 | 59,1 | 29,84 | 29,72 | 29,78 | 7 | 15 | 9 | 0 | 0 | 0 |
| An. mean. | 60,7 | 67,9 | 72,4 | 29,84 | 29,74 | 29,70 | 95 | 166 | 93 | 28 | 9 | 2 |

| 1838. | *Thermometer.* 6 A. M. | 12 M. | 6 P. M. | *Barometer.* | | | cl.days | cl'dy. | rain | thaw | fog. | snow. |
|---|---|---|---|---|---|---|---|---|---|---|---|---|
| January, | 47,6 | 52,2 | 55,6 | 30 | 30, 2 | 30, 5 | 5 | 18 | 8 | 1 | 0 | 0 |
| February, | 37,6 | 41,9 | 47,6 | 29,92 | 29,91 | 29,87 | 4 | 12 | 7 | 3 | 2 | 5 |
| March, | 55,6 | 60 | 66,8 | 29,79 | 29,71 | 29,73 | 2 | 9 | 9 | 3 | 14 | 0 |
| April, | 61,8 | 71,6 | 74,6 | 29,74 | 29,74 | 29,70 | 2 | 19 | 4 | 3 | 5 | 0 |
| May, | 62,2 | 70,2 | 73 | 29,66 | 29,69 | 29,67 | 1 | 17 | 13 | 3 | 0 | 0 |
| June, | 72,6 | 85,5 | 88,1 | 29,69 | 29,73 | 29,67 | 1 | 27 | 2 | 1 | 0 | 0 |
| July, | 76,4 | 82,5 | 85,1 | 29,80 | 29,77 | 29,79 | 0 | 19 | 12 | 10 | 0 | 0 |
| August, | 74,3 | 83,6 | 84,5 | 29,75 | 29,76 | 29,71 | 0 | 19 | 12 | 13 | 0 | 0 |
| September | 66,5 | 76,2 | 78,9 | 29,68 | 29,72 | 29,71 | 10 | 14 | 6 | 2 | 0 | 0 |
| October, | 56,5 | 65,3 | 65,4 | 29,80 | 29,80 | 29,79 | 8 | 15 | 8 | 1 | 0 | 0 |
| November | 46,2 | 56,8 | 55,3 | 29,88 | 29,89 | 29,87 | 5 | 11 | 7 | 2 | 7 | 0 |
| December | 37,9 | 46,2 | 50,2 | 30,03 | 29,93 | 29,91 | 5 | 13 | 7 | 0 | 3 | 5 |
| An. mean, | 57,9 | 66 | 68,8 | 29,89 | 29,89 | 29,79 | 93 | 193 | 92 | 42 | 31 | 10 |

| 1839. | *Thermometer.* 6 A. M. | 12 M. | 6 P. M. | *Barometer.* | | | cl.days | cl'dy. | rain | thaw | fog. | snow. |
|---|---|---|---|---|---|---|---|---|---|---|---|---|
| January, | 43,4 | 52,7 | 58,8 | 30,02 | 29,97 | 29,88 | 0 | 21 | 10 | 2 | 0 | 0 |
| February, | 45,5 | 51,9 | 55 | 29,99,7 | 30,10 | 29,98 | 5 | 13 | 10 | 0 | 0 | 0 |
| March, | 53,4 | 59,7 | 64,8 | 29,90 | 29,91 | 29,90 | 2 | 19 | 8 | 2 | 2 | 0 |
| April, | 68,9 | 74 | 82 | 29,73 | 29,71 | 29,67 | 2 | 25 | 3 | 2 | 0 | 0 |
| May, | 68 | 80 | 81 | 29,71 | 29,73 | 29,70 | 4 | 20 | 7 | 7 | 0 | 0 |
| June, | 74,8 | 84 | 88 | 29,77 | 29,78 | 29,78 | 0 | 26 | 4 | 4 | 0 | 0 |
| July, | 75,4 | 86,5 | 89,6 | 29,69 | 29,70 | 29,70 | 0 | 23 | 8 | 5 | 0 | 0 |
| August, | 74,2 | 84,5 | 86,5 | 29,73 | 29,74 | 29,71 | 2 | 23 | 6 | 7 | 0 | 0 |
| September | 68,7 | 79,8 | 82,7 | 29,80 | 29,80 | 29,78 | 0 | 23 | 3 | 1 | 4 | 0 |
| October, | 63,8 | 74 | 77 | 29,82 | 29,82 | 29,50 | 0 | 20 | 3 | 0 | 8 | 0 |
| November | 48,3 | 54,2 | 55,7 | 29,01 | 30,89 | 29,60 | 3 | 14 | 10 | 1 | 3 | 0 |
| December | 44,3 | 47,2 | 49,1 | 29,89 | 29,78 | 29,82 | 8 | 9 | 9 | 0 | 5 | 0 |
| An. mean, | 61,1 | 69,1 | 72,5 | 29,82,5 | 29,82,5 | 29,69,9 | 26 | 235 | 82 | 31 | 21 | 0 |

The measles prevailed during the winter and spring of 1836; the excitement was open and where treatment was necessary, it generally yielded to emetics of calomel and ipecac 8 or 10 grs. each, followed by gentle laxatives of Epsom salts and senna, or calc. magnesia, anodyne sudorifics of Dover's powder, or paregoric, and ipecac wine, at night, with the free use of mucilaginous drinks.

A few cases terminated in pneumonia, or acute bronchitis requiring depletion to the extent of making a decided impression on the system or relieving pain and difficulty in breathing, assisted by mild laxatives, blisters, and, if not speedily relieved, alterative doses of calomel and ipecac, repeated every second, third or fourth hour, until the secretions of the liver and mucous membranes improve or a slight ptyalism commences. The combination of Coxe's hive syrup and paregoric, was used as an expectorant, given in elm, flaxseed or gum arabic mucilage. In a few enervated habits, the eruption did not develope itself, or it receded suddenly, with general coldness of the surface, livid complexion, difficult respiration and great restlessness. In such cases, stimulating baths of mustard, or frictions with pepper or mustard in hot brandy, were advised, and full portions of camph. vol. alkali and calomel were repeated as circumstances indicated. The compound which I employed was the following:

℞. camph. and vol. alk. each 5 grs., calomel 5 to 10 grs. mixed, to be given every 2 or 3 hours. As a drink, the decoction of polyg. senega or warm toddy, until the excitement was diffused, or the symptoms were relieved.

Many who had been previously affected with measles, had slight catarrhal fever.

The treatment of bronchitis with the exception of blisters to the throat or neck, and mild gargles, differed but little from that of pneumonia.

Influenza occurred late in the spring amongst the blacks on a few plantations, of quite a malignant character—cough, sore chest, pain in the head and back, a white or blue furred tongue with red edges, and moderate thirst. In most cases there was no chill, action of the pulse moderate, breathing difficult; but in some, the excitement seemed crippled, and irregularly diffused, extremities cold, and the stomach irritable with great nausea and restlessness, and after the third or fourth day sub-inflammation of the whole mucous membrane with diarrhœa frequently closed the scene.

In the open form of the disease, moderate depletion, emetics of cal. and ipecac. or lob. inflat. laxatives, alterative doses of calomel and ipecac, counter irritants to the chest and extremities, expectorants, and Dover's powder at night, generally relieved. In many cases, regardless of the *endemic* character of the disease, the patients were either suddenly prostrated by a single large bleeding, or under frequent small bleedings, drastic purges of calomel, aloes, and scammony or jalap, and premature blisters, generally sunk into a typhoid state, with engorged lungs, and rattles, and died about the sixth or eighth day.

A mild remittent, and intermittent fever, with griping and loose bowels, (in those who had fever during the previous fall) succeeded the influenza and yielded to the usual remedies.

From 1st of May until July, there was little disease, some cases of bowel complaint in children, with consecutive determination to the head, ending in spasms. Emetics of calomel and ipecac, 4 grs. each, followed by minute portions of the same as an alterative, with mucilage, generally cured—leeches or cups to the abdomen or head, were very useful to some.

July was more sickly, and early in the month, there were some severe cases of fever on the flat lands with great determination to the head; general and local depletion, the cold douche, with brisk mercurial cathartics relieved most of the cases. Coughs and bowel complaints were common amongst children, and there were a few cases of chronic dysentery the sequelæ of measles.

On the high-land plantations there were a few cases of malignant double tertians, and more sickness in town than usual; diarrhœa, dysentery and whooping cough amongst the children—fever amongst grown persons quite obstinate and attended with severe hiccough. Bleeding and mucilaginous drinks relieved the head, the hiccough and the heavy exhalation from the skin and bowels, calomel restored the healthy secretions, and as soon as there was a sufficient remission, quinine in from 2 to 5 gr. doses, every second hour completed the cure.

The diseases in August did not vary either in form or violence from those of July.

In September, disease increased considerably, in the form of remittent fever, dysentery and frequent cases of malignant double tertian, the severe paroxysm commencing at 10 o'clock, A. M., and the light one at 4 P. M., on the following day. Some died in the first cold stage sinking immediately into fatal collapse, and but few survived the third chill, unless it had been preceded by very prompt and active treatment. In most cases, the stomach and bowels took on choleric action ; some after one or two convulsions, sunk into profound coma, and in every case when there was any show of reaction, it was feeble, irregular, and terminated in an icy coldness of the surface, the tongue moist, very pale and clean, or covered with a blue moist fur, and the thirst moderate.

This form of disease I fear has been too often mistaken (in some parts of this State) for Dr. Armstrong's congestive typhus and treated accordingly. They are antipodes, so far as respects the pathological condition of the organs, but I shall decline saying any more on the subject it being rather irrelevant.

In the above form of disease, where the patient was seen in the first paroxysm of torpor, if there was some effort to react the remedies which were used with most success, were emetics of calomel and ipecac, followed by large alterative doses of the same, at intervals of one, two or three hours, until secretion was procured from the bowels, with frictions perseveringly applied to the extremities, and warm drinks until reaction was established. Whenever the action began to decline, full doses of quinine every second or third hour, alternated with ten grain doses of calomel, with sinapisms of mustard to the extremities, prevented a return of the chill and torpor. The medicines were then continued in smaller quantities, and at longer intervals, until the secretions became natural and the patient convalescent. The following is my mode of administering the quinine:

℞ sulph. quin. grs. x.

Pip. nig. ol. grs. i. M. ft. pil.

One to be taken every hour, or once in two hours.

When the patient was not seen before the second chill, the extremities were icy cold, stomach very irritable, contents of the bowels liquid ; a warm mustard, pepper or salt bath or mustard sinapisms to the stomach and extremities, blisters, quinine as above prescribed alternated with calomel twenty grains, sulph. morph. 1-6 grain, together with warm stimulating drinks, seemed to snatch the patient from the jaws of death, and relieved him, contrary to the expectations of friends or physician. Injections of strong salt-water were used to empty the bowels when necessary.

Many auxiliaries were tried but seemed of no effect—a slight ptyalism sometimes supervened but rather expedited than retarded the cure; convalescence was speedy and permanent.

To many readers it may seem, that too much has been said on this part of the subject, but it is one of the first importance to the southern practitioner, and although some may say that less calomel and quinine would have relieved the patient, I found from ample experience, that there was no time to be lost, neither should there be any scruples about doses.

The blacks suffered more than the whites, probably from two causes, viz: the want of timely aid, and the more enervated state of the system.

There was more disease amongst the acclimated than there had been since 1831.

After severe frost, on the 20th, 21st and 22d of October, disease was more of the remittent character and bore the lancet and antimonials with good effect.

There were a few cases of complicated rheumatic states of fever in the course of the month.

But little disease prevailed in November, until the 15th, when the influenza commenced; at first very mild, but it gradually increased in violence, assuming the type of pneumonia, with severe pain in the chest, hard cough; in the worst cases the expectoration was white, thin and frothy, in the more mild it was of an olive cast, sometimes much mixed with blood; there was also oppressed breathing, either very torpid or loose bowels, high arterial excitement, white or yellow furred tongue and some thirst. Amongst the white population it required the same treatment as in the previous spring—amongst the blacks it seemed to produce sudden prostration, assuming all the phenomena of typhoid pneumonia, and requiring the treatment heretofore mentioned.

There were during the winter and spring, many anomalous symptoms, such as pain and suppuration of the ears, and of the frontal sinus, tooth ache, inflammation and suppuration of the cellular membrane, together with diarrhœa, and dysentery, all, it is very likely, dependent upon the peculiar epidemic constitution of the atmosphere.

In February there were a few cases of tonsilitis, some scarlatina, also small-pox of the distinct kind—a few of the cases were pronounced varioloid, but there seemed to be no marked distinction in them, or in the treatment required—only two or three died.

During the spring, anasarca prevailed amongst the unacclimated blacks—it generally yielded to one moderate bleeding, cathartics of jalap, or gamboge, and cream of tartar, alterative pills of calomel and squill, and the tincture of muriate of iron.

From April to August, the city and country were more exempt from disease than they had been for many previous years.

### THE DISEASE OF 1837.

This has, by common consent, been called "yellow fever," but it frequently ran its course, either terminating in death or recovery, without the appearance of yellowness of the skin or eyes, which was not therefore a distinctive character of the disease.

It has been called "black vomit," but many cases proved fatal unaccompanied by that symptom, neither was that fluid found in the stomach on dissection.

It was not as some have supposed, a high or malignant grade of bilious fever—the whole surrounding country being more exempt from that disease than usual, (particularly in 1839,) there being almost an entire absence of moisture, one of the principal agents in the generation of miasma—also in most cases of recovery, the first satisfactory evidence of amendment was the plentiful secretion of a dark acrid bile, which in many instances excoriated the rectum in its passage.

It was not any of the modifications of typhus, and there was no evidence on dissection of any affection of the mesenteric or other glands.

In no case of dissection did Dr. Pollard find any degeneration of the glandular system.

Dr. Pollard saw four cases in the hospital of evident congestion, two on the first day which carried off the patients in five or six hours, the other two died later but proved of the same nature, without hiccough, black vomit or any signs of inflammation. He saw others in whom there was complete prostration of all the powers of life, without his being able to trace the disease to any important organ, the patient dying in a few hours.

It was not essential gastro enteritis, there being many cases of death without any appreciable symptom from which inflammation could be suspected, neither did dissection shew anything to justify such conclusion.

If it were neither of the foregoing diseases, the question necessarily arises—what was it? I must briefly answer that it was an assemblage of symptoms or rational signs, indicating the modified condition of such organs, as previous predisposing causes might have made obnoxious to the morbid influence of a specific cause, not cognizable by our senses, a poison sui generis, infecting a certain portion of atmosphere. A disease produced by such a cause, must be sui generis, proving fatal in many cases without the intervention of active inflammation, or the patient becoming gradually convalescent without any of the critical phenomena that could enable the most acute observer to form a correct prognosis as to the result.

The disease, after a few hours of listlessness and indisposition to exercise, commenced with a well marked chill, succeeded by free open reaction, frequent, full, resisting pulse, pain in the head, back and limbs, red eyes, restlessness, watching and frequently delirium, terminating in profound coma before death, considerable thirst, moist, clean, or slightly furred tongue—irritable stomach, with soreness about the pyloric orifice on pressure, and in a few hours great nausea and vomiting. The bowels were generally slow, discharges of a light grey or of a blue muddy cast; the skin was commonly hot and dry, and when yellowness attended, it generally began about the second or third day, and in fatal cases, gradually increased until death—in the cases of recovery it subsided as the secretions improved. In nearly every fatal case it became yellow after death. The eyes were seldom yellow.

When black vomit was present, it usually began from twenty-four to forty-eight hours before death, and was in most cases accompanied by hiccough—the mucus which had been previously thrown up clear and mixed with small white flocculi, began to show small black specks mixed with it, rapidly increasing until it assumed its specific character, and was in most cases belched up without any effort of the patient. In some of the hospital cases the same kind of matter was discharged per anum. Hiccough was frequently distressing when there was no black vomit.

Hemorrhage was not frequent, but when it occurred from the nose, it was generally favorable—when from the mouth or bowels it was in most cases fatal—the urine was scanty and high colored, and sometimes very dark, resembling black vomit. Changes from dry to cold damp weather were very unfavorable, most of the severe cases proving fatal, and most of the convalescent relapsed unless they were very prudent. There was a suspension of new cases for a few days, late in October, which induced numbers to return to the city who had left it, and put those off their guard who had remained. Very soon, however, the disease recommenced, and there were a number of deaths.

Males were more frequently attacked, and the disease was more unmanageable in them, than in females or children. The black population suffered least.

The young, stout, robust, and intemperate were most obnoxious. The acclimated were generally exempt, but there were a few cases amongst those who had had the disease in other places, and none of them proved fatal.

The first death in a well marked case, was on the 7th of September, nearly in the centre of the city, where the disease first appeared—there were but few cases under the Hill—the last death was on the 4th of December, and the whole number of deaths was 257.

The population of the city at the commencement, exceeded 6000, and but few left it during the prevalence of the disease.

Something may be expected on the subject of prognosis, but such was the peculiarity of the disease, and so insidious were the approaches of death, that the most experienced observer would be deceived; in fact, there were none of those critical circumstances attending most other diseases, and the remissions were very short and obscure. For several days, the organs seemed to resist and throw off its influence in proportion to their recuperative powers, the stomach and skin being generally the last to resume their normal functions. After a change of weather and two or three frosts the disease subsided.

In the *commencement* of this as of most other malignant endemics, many died of the number severely attacked, although few of the Faculty acknowledged the loss of patients. The treatment of former seasons did not succeed—antimonials as emetics, or to answer other indications, not only failed, but in many instances seemed to do evident injury.

In the forming or early stage of the disease, after prompt depletion, in which the blood was florid, sometimes the the emetic of calomel and ipecac, followed by castor oil, or senna tea seemed to check its progress, softening the pulse and skin, relieving the head and promoting the secretions, and if judiciously assisted by local depletion from the head, spine or region of the stomach, by counter irritant and alterative portions of calomel or blue mass and ipecac or James' powder, with drinks of cold mucilage, ice water or lemonade, the chance for the patient's recovery seemed better than from any other plan. Both general and local bleeding was freely used in the hospital by Dr. Pollard with decidedly good effect.

Many auxiliaries were used as palliatives, to allay the irritation of the stomach, and to promote the specific secretions of the skin, kidneys, &c., but the ice, mucilage, milk and lime water, soda and charcoal, were the only ones which seemed to prove useful. In the hospital the oil of amber seemed to have a good effect, particularly in the only case of black vomit in which recovery took place. Seidlitz powders, castor oil, or senna tea, were useful laxatives after the secretions improved; unless the patients was much prostrated. Injections of warm water, gruel or salt water were used in that event.

During the insidious respite, or remission of the disease which frequently occurred on the third or fourth day, quinine combined with calomel, blue mass, and camphor, or oil of black pepper, and other stimulants, was tried by some, and thought very beneficial, whereas in the opinion of others, it seemed not only unprofitable, but evidently injurious, the disease again recurring with increased violence, black vomit coming on and soon closing the scene. Dr. Pollard informs me that most of the patients taken to the hospital in the early stage of the disease, who had been previously treated with calomel and quinine without free depletion had raving delirium, red eyes, hot skin and great thirst, and, if in the advanced stage, they had low muttering delirium, dry brown, sharp pointed tongue, which they protruded with difficulty. The head was very hot and there was acute pain in the stomach, but seldom any black vomit. When there was free depletion, and calomel was combined with the quinine, it was not so injurious, but he thinks did no good.

The "post hoc, ergo propter hoc" experience in the profession has no doubt in this, as in many other diseases, done much mischief, for every candid practitioner must acknowledge that he has frequently seen cases of recovery where the recuperative powers of the system have resisted the most preposterous prescriptions, and his after and more correct experience, has made him blush for his previous ignorance.

After the subsidence of the epidemic, the city and country were very healthy, and such disease as prevailed, did not as usual seem to wear its livery in the slightest degree. From December, 1836, up to August, 1837, there was a period of severe trial with the faculty, almost amounting to starvation. The alarm was then again sounded, and the second epidemic commenced.

### EPIDEMIC OF 1839.

The first case of this disease was taken to the Hospital from "under the Hill," but it soon became general; the first death was on the 27th of August, and the last on the 20th of November, and the whole number reported by the sexton, was 235, a few died who were taken to the country, and in two instances others of the family where they were, had the disease who had not, from their statement, been within the circle where it prevailed. The population of the city at the commencement, did not exceed five thousand—many left the city immediately, and by the first of October, the number was not greater than 1500.

The acclimated and the blacks suffered more than in 1837—many were attacked who had previously had the disease, a few of whom had it severely, but there were not many of them who died.

The attack was more insidious than in 1837; many seemed quite exhilarated and boasted of their good health, but a few hours before they were attacked; but in most cases, there was great depression of spirits, indifference to surrounding objects and a horror which seemed gradually to increase, until there was a slight coldness of the hands and feet, and a sensation of chilliness down the spine—this condition was indefinite, lasting from a few minutes to several hours, before the succeeding phenomena were developed.

In the mild cases, the excitement was free and well diffused, pulse varying from eighty-five to one hundred in a minute, full, firm and resisting—the skin hot and dry and seldom yellow—pain in the head, back and limbs moderate—some restlessness and watching, but rarely delirium—the tongue was moist and clean, or covered with a thin white fur—considerable thirst; after a few hours the stomach became irritable and sore to the touch, succeeded by frequent vomiting of a fluid differing but little in character from that of 1837—the bowels very slow, the urine scanty and high colored.

In such of those cases as proved fatal, the soreness of the abdomen, vomiting, obstinacy of the bowels, and thirst increased, the secretion of urine was entirely suspended and death closed the scene, frequently without the appearance of black vomit.

Hiccough was common, and in some cases there seemed to be a morbid secretion of gas by the stomach more distressing than the vomiting.

In the more violent form of the disease there was no appreciable chill—the skin was moderately warm or cool, and of rather a leaden hue, the excitement was irregular and crippled, pulse varied from one hundred to one hundred and thirty, with throbbing of the carotids, shrunk features, cold or cool extremities, little or no pain, great oppression of the stomach with soreness to the touch in some cases, the tongue was moist, pale, expanded, with a pale blue fur and red edges, thirst and distressing vomiting, the bowels even obstinately costive, and when evacuations were procured, they were of a dark or blue muddy cast, or liquid and almost colourless. The urine was scanty, thick and dark, resembling black vomit, or it was totally suspended.

In some of those cases black vomit came on very early, and death occurred frequently within the first three days—many seemed to die without the signs of inflammation being present, and evidently from the exhaustion of the nervous influence. In the last named cases there was frequently no black vomit. The skin became invariably yellow after death.

Hæmorrhage occasionally came on, and when from the nose it was favorable, but when from the mouth, bowels or kidneys it was always unfavorable and most frequently the precursor of death. It seemed to be the effect of calomel in but one case that came to my knowledge. In the hospital practice both in 1837 and 1839, where the patient was not freely bled in the early stage and mercury was given so as to produce salivation, it was generally severe and frequently brought on hemorrhage from the mouth or bowels, and the convalescence was slow and imperfect—when he was freely bled the calomel answered every purpose as a cathartic and there was no necessity for it as an alterative, or for quinine.

The disease from the 16th of November, became gradually milder and ultimately subsided without any remarkable change in the weather.

The rational signs of improvement whereby the physician could form his prognosis as to the probable event of the disease, were more obscure than in 1837—the convalescence was gradual and progressive without any specific crisis.

I saw but one sporadic case—it occured in February, the patient had been sick six days and was mortified, death taking place in ten hours after I saw him. In this case there was no symptom indicating to me, the approach of death—the mind was calm, pulse open, soft, and eighty strokes in a minute—skin warm and moist, stomach quiet, but the bowels liquid.

No prophylactic except temperance in all things seemed to prevent the disease—calomel was relied on by some, but several were attacked when under its influence, and some died when highly salivated although it was generally favorable.

### TREATMENT OF THE DISEASE IN 1839.

In the first cases which occurred, the attack was so insidious, the deaths so frequent, under circumstances which, when present in the ordinary fevers of the country, would not warrant such a result, that the faculty were astounded, the firmest of them were thrown off their balance, and it became a perfect matter of experiment with them. Some gave antimonial emetics, and as an after treatment, relied upon cupping, mild laxatives, mucilage, counter irritants, and diaphoretics—some bled moderately, cupped, gave no cathartics, used injections, counter irritants and diaphoretics, palliatives to relieve the stomach, and quinine—others gave strong mercurial cathartics, followed by alterative doses of calomel, camphor and quinine, stimulating diaphoretics, with warm teas: many other plans were tried not necessary to notice.

After the panic had somewhat subsided and reflection and reason resumed their empire, the faculty began to take a different view of the disease, and the most successful treatment so far as I saw and was able to learn from the very intelligent physicians of the place, was a judicious modification of the following outline:

In the mild form, when the reaction was free and open, one full general bleeding, (the blood generally florid or of a scarlet red,) had a very happy effect, in allaying the pain, thirst, irritation of the stomach and obstinacy of the bowels—this was usually followed by a full dose of calomel alone, or combined with ipecac or James' powder, and if it failed to operate as a cathartic in six or eight hours, castor oil, senna or some other laxative was given to promote its operation. Cupping followed by mustard sinapisms to the stomach, gave great relief—ice water, mucilaginous lemonade, or cold toast water, taken in small quantities, generally allayed thirst, and a few alterative doses of calomel or blue mass, either alone or combined with camphor and quinine, improved the secretions, modified the succeeding exacerbation and seemed so far to disarm the disease, that the after treatment, consisted of attentive nursing, an occasional Seidlitz powder, castor oil, magnesia, or injections, to regulate the bowels, with an occasional anodyne sudorific at night. Some continued the alterative until gentle ptyalism commenced, and there were but few cases in which it was excessive or proved injurious.

In the more violent or irregular congestive form of the disease, the first and all important object was to diffuse the excitement, relieve the oppressed organs, and enable the brain and nervous system to supply and regularly distribute a sufficient quantity of nervous influence, to excite the secreting organs to resume their functions, and thereby prevent the sudden and unaccountable deaths, or the permanent local congestions, which either quickly resulted in active inflammation with its consequences, or became a focus of irritation, which although more protracted in its influence generally proved fatal.

There were two plans adopted to fulfil the necessary indications, each of which had its advocates amongst the most intelligent of the profession, both claiming a reasonable share of success as the result; but as the illustrious Sydenham has said "we do not always cure disease in the best way."

The first was (in the early or forming stage of the disease) to administer full and frequent doses of quinine combined with blue mass or calomel, and camphor, oil of black pepper, or other diffusive stimuli—cupping the region of the stomach, sinapisms of mustard to the extremities, frictions and external warmth, continued until the action was diffused; after which if any important organ was threatened, or other circumstances indicated, general bleeding was resorted to, together with alteratives, laxatives and other remedies, as advised in the mild or open form of the disease.

The second plan was to detract blood, in small quantity, and repeat it according to circumstances, in the forming stage. The blood was generally of a dark modena cast and difficult to obtain, but after reaction was developed it was very florid and buffy. In one or two cases it had a peculiar fœtor very offensive to those who were present. In the hospital depletion was resorted to in frequent small quantities to procure reaction with the happiest effect. To this succeeded the administration of calomel and ipecac, so as to make a moderate impression on the stomach; this was followed, in a few hours, by laxatives of calomel, magnesia and rhubarb, salts and senna, castor oil or Seidlitz powders; cupping the region of the stomach or skin which was always useful: sinapisms to the extremities and warmth were likewise employed to the surface; and when the reaction became fully established one full general bleeding. This allayed the gastric irritation and thirst, and prepared the organs to resume their functions under the administration of the proper secernents. Blisters were sometimes used, but were rarely beneficial, producing severe strangury, which nothing seemed to palliate. Patients, indeed, frequently died under its influence. Magnesia in sweetened water, carbonate of soda, ice water, or milk and lime water, were the best palliatives in irritable stomach—charcoal was used, but without any marked advantage. In the hospital the oil of amber seemed to answer better than any other remedy. Relapses were more frequent than in 1837, and in most cases fatal.

A very fine white vesicular or miliary eruption sometimes appeared about the neck and chest, and was always a fatal symptom.

Very dark vitiated evacuations from the liver and mucous membranes of the bowels were generally the first well marked evidences of improvement—these were followed by such as were of a more healthy cast, and in some cases continued for several days before the gastric irritation entirely subsided.

I am not prepared to treat of the ætiology of this fever. Its pathology may be inferred from the symptoms, the effects, as detailed, of different modes of treatment, and the autopsic examinations of my intelligent young friend, Dr. Pollard, (resident physician of the Hospital,) which are annexed to this paper.

All the bodies examined after death, were those in the hospital. They had generally been paupers, of loose, intemperate habits; most of them young or of middle age, whose organs no doubt, from previous habits, were predisposed to congestion, or to active inflammation from the application of any exciting cause, and many of them in 1837, had not been depleted during their out-door treatment, but had taken freely of calomel and quinine: hence some allowance ought to be made for the frequent extensive structural lesions found in the organs, many of which might not have taken place under different circumstances.

I shall give Dr. Pollard's Report as handed to me by himself.

"Dissections were generally made four hours after death, by myself assisted by Dr. T. C. Lawrence, both in 1837 and 1839. The skin became yellow, after death, when it was not previously so; the back and under parts of the limbs, placed in a horizontal position, were of a dark red and sometimes ecchymosed appearance. On opening the head in those who had been bled, or the disease had been suffered to run an uninterrupted course, the dura mater was natural, the tunica arachnoides and substance of the brain were natural; in some of those cases, a tea-spoon full of serum was found in the lateral ventricles. The lungs were natural, filled as much as common with air, and with blood collected in their lower parts as they lay. The substance of the lungs was generally healthy. In the abdomen, the stomach, when laid open, was found to have undergone some alteration in almost every case; red patches were to be found in some, as large as a dollar, in others smaller; they were generally about the pyloric orifice, but rarely in the cardiac portion. The organ in most instances, contained nothing except what had been administered to the patient.

The red spots were also very generally to be found in the duodenum. The other bowels presented a healthful appearance.

The liver was paler than usual, the gall-bladder contained a small quantity of green bile. The spleen was also pale. The kidneys were generally healthy, but in three or four cases they contained mucus.

I observed that in those who had not been bled or who had taken quinine, the brain had suffered. The arachoid was engorged, and the ventricles almost always contained an excess of serum. On making a section into the substance of the brain, small points of blood appeared in numbers. The red spots on the mucous membrane of the stomach and duodenum were large and numerous. In those who died of black vomit, sometimes the same was found in the stomach and bowels, but the mucous membrane was entire, only showing the marks of inflammation. These observations were made in 1837.

The cases that I examined in 1839, presented pretty much the same features in the stomach and liver, only in a more aggravated form. The brain this year was seldom altered from its natural character. In some two or three, serum was contained in the lateral ventricles. The lungs in many were filled with blood, and the auricles of the heart, particularly engorged. The pericardium contained more liquid than is natural. The stomach and duodenum were highly inflamed (except in the cases to be mentioned,) the mucous membrane in some was softened and covered with a viscid mucus. The liver was pale and the ducts filled with mucus, The gall bladder seldom had much bile in it and in some it was filled with a gelatinous mucus. The bowels, with the exception of the duodenum, had no traces of disease, even in those who had voided blood, except that the membrane was softened.

The bowels contained sometimes blood, and sometimes mucus. The kidneys were also filled with mucus which would run from them when squeezed, as from a sponge filled with water.

In the cases that died from want of reaction in the commencement, I could observe no very marked diseased condition. The brain looked quite healthy, and also the viscera of the chest. The stomach was slightly altered about the lower orifice, small red points were seen, which were also to be perceived in the duodenum. The kidneys in these cases were natural. The mucous membrane of the bladder was healthy in all the subjects."

